# Air-filled SIW technology for mass-manufacturable and energy-efficient terahertz systems

**DOI:** 10.1038/s41598-023-43887-0

**Published:** 2023-10-04

**Authors:** Laura Van Messem, Siddhartha Sinha, Ilja Ocket, Heinrich Trischler, Erich Schlaffer, Daniel Schlick, Hendrik Rogier, Sam Lemey

**Affiliations:** 1 IDLab, Ghent University, 9052 Ghent, Belgium; 2 IMEC, 3001 Leuven, Belgium; 3 AT&S AG, 8700 Leoben, Austria

**Keywords:** Electrical and electronic engineering, Characterization and analytical techniques

## Abstract

To accommodate the ever-growing data requirements in densely populated areas and address the need for high-resolution sensing in diverse next-generation applications, there is a noticeable trend towards utilizing large unallocated frequency bands above 100 GHz. To overcome the harsh propagation conditions, large-scale antenna arrays are crucial and urge the need for cost-effective, mass-manufacturable technologies. A dedicated Any-Layer High Density Interconnect PCB technology for highly efficient wireless D-band (110–170 GHz) systems is proposed. Specifically, the adapted stack accommodates broadband air-filled substrate-integrated-waveguide components for efficient long-range signal distribution and low-loss passives. The viability of the suggested technology platform is demonstrated by designing, fabricating and measuring several essential low-loss air-filled substrate-integrated-waveguide components, such as a dual rectangular filter, with a minimal insertion loss of 0.87 dB and 10 dB-matching within the (132.8–139.2 GHz) frequency band, and an air-filled waveguide with a routing loss of only 0.08 dB/mm and a flat amplitude variation within 0.01 dB/mm over the (115–155 GHz) frequency range. A broadband transition towards stripline, with a limited loss of 1.1 dB, is described to interface these waveguides with compactly integrated chips. A tolerance analysis is included as well as a comparison to the state of the art.

## Introduction

Adding sensing capabilities to the future communications network will greatly improve overall safety in a wide variety of situations and will facilitate reliable spatial signal distribution. To this end, joint communication and sensing (JCAS) in 6G networks enables massive machine-to-machine (M2M) communications, for example on the road for object detection and traffic monitoring, or on a factory ground to ensure machine-safety for operators^1,2^. The increasing resolution for next-generation radar systems and the growing data demand, both in terms of bandwidth and data rate, which originate from the aforementioned paradigm, force technology to explore the higher sub- and terahertz frequency spectrum where large chunks of the spectrum are still unallocated. Beneficial atmospheric properties, even in rainy conditions, favor D-band (110–170 GHz) frequencies for emerging systems^3^. The latest research achieves datarates up to 150 Gbps in this band, resulting in a hundredfold throughput increase compared to our current network speed^4^.

The deployment of antenna arrays to overcome increased path loss when upscaling to higher frequencies introduces its own challenges^5–8^. On the one hand, the required power handling capability for future systems, next to low-loss routing towards the antenna elements, advocates air-filled substrate-integrated-waveguide (AFSIW) transmission line technology^9–11^. Cost-effective mass-manufacturability and integration prospects of active electronics, on the other hand, restrict the fabrication technology further. To this end, an elaborate comparison between existing technology platforms is discussed and summarized in Table 1. The most traditional high-precision fabrication process with excellent repeatability is computer numerical controlled (CNC) milling. The resulting prototypes are commonly constructed out of aluminum or a steel alloy, which may be gold plated to improve the conductivity and avoid potential detrimental effects of the magnetic properties of the base material^12–17^. Interfacing monolithic microwave integrated circuits (MMICs) proves to be quite cumbersome, while manufacturing is done sequentially and remains expensive for high-accuracy parts, reducing the scalability towards mass-production. Excellent surface roughness is achieved by polishing at the cost of an expensive time consuming process. A low-cost, chemical metal etching process alternative^10^ with similar insertion loss has been proposed to tackle equivalent hurdles. However, the prospects of MMIC integration remains an obstacle. The stereolithograpy (SLA) and direct metal laser sintering (DMLS), also called micro metal laser sintering ($\mu$MLS) or selective laser melting (SLM), 3D-printing technologies provide an alternative to counter the material waste of milling^18–24^. In contrast to DMLS-prints, the intermediate SLA resin samples have to be plated before a functional prototype is obtained. The feature size and surface roughness are mainly limited to the particle size, in case of DMLS, and to the laser properties, in case of SLA. Injection molding provides a scalable alternative for high volume productions. Promoting this technology for commercial future terahertz systems requires solving the current pitfalls of demoulding defects and the limited aspect ratio^25–28^. Excellent feature size and aspect ratio are obtained with SU-8 and silicon micromachining fabrication. Where mechanical stability and poor thermal properties constitute the main drawbacks of SU-8 technology^13,29–32^, a pitfall of the silicon micromachined waveguide (SMW) process is the cost^14,33–38^. For both approaches, compact MMIC integration is very challenging due to the presence of silicon, leading to high interface losses and reduced bandwidth^11^, and the additional need for dense networks to distribute DC and control signals to all MMICs^4^. Furthermore, low temperature co-fired ceramics (LTCC) technology allows development of (partial) dielectric antenna topologies, although at the cost of high losses due to its high relative permittivity^39–41^. The limited thickness of the ceramic substrates requires stacking multiple layers, making the process prone to fabrication defects, warpage and alignment errors. Finally, multiple printed circuit board (PCB)-based D-band multi-antenna systems have been proposed^5,8,42^. While promising results are obtained, they suffer from considerable routing losses due to the use of conventional transmission line technology, leading to a degraded noise figure at the receive side, reduced effective isotropic radiated power (EIRP) at transmit side and a restriction on the amount of supported antenna elements.

In this paper, we present an innovative cost-effective PCB-based technology platform that enables the implementation of highly efficient AFSIW-based routing and compact integration of active electronics. Two transitions, one from grounded coplanar waveguide (GCPW) to stripline, and, one from stripline to AFSIW, are designed to access the low-loss AFSIW section. In this region, transmission lines of different lengths and functional components, such as a filter, are designed in AFSIW to validate the proof of concept. Comparing different line lengths allows extracting the measured transmission loss, which remains around 0.08 dB/mm in the entire D-band. Furthermore, the stripline-to-AFSIW transition exhibits 1.1 dB-loss, characterized by comparing the different calibration planes for a single line length. The proposed two-cavity filter showcases the reliability of the fabrication process for more complex AFSIW components. This component has a footprint of 5.2 mm $\times$ 2.2 mm $\times$ 300 ${\upmu }$m, a minimal measured insertion loss of 0.9 dB at 136 GHz, with a 10 dB impedance matching bandwidth ranging from 132.9 to 139.2 GHz and a group delay smaller than 0.07 ns. The main characteristics of this novel technology platform are compared to existing state-of-the-art D-band technology platforms in Table 1, including only the best results employing optimal fabrication processes for the latter. By eliminating dielectric losses, the employed AFSIW layer allows significant reduction of the insertion loss w.r.t. Any-Layer High Density Interconnect (HDI) PCB and LTCC technology. It can be seen that our proposed PCB technology platform with a dedicated AFSIW layer offers a low-cost, mass-manufacturable alternative at D-band frequencies that facilitates compact MMIC integration versus CNC milling, ($\mu$MLS), injection molding, SU-8 or SMW, paving the way for future cost-effective, highly integrated wideband and low-loss JCAS systems^43,44^.

Leveraging an air substrate for the implementations of high-efficiency microwave components in PCB technology, such as filters, beamsteering networks and antennas, is well established at sub-6 GHz and lower millimeterwave frequencies^45–49^. These proposed solutions make use of cheap standard PCB manufacturing technology and achieve their multi-layer stack-up by manually aligning and fixing with screws. This approach is cumbersome for beyond 100 GHz frequencies. Therefore, we suggest to evolve towards multi-layer PCB manufacturing to avoid manual alignment, screws or solder. In this respect, the significant routing losses of the current PCB implementations at D-band (110–170 GHz) highlight the importance of adding a highly efficient AFSIW routing layer, as introduced in our proposed solution. This article extends previously published work^50^, by discussing the technology platform in more detail, performing an in-depth tolerance analysis and validation of fabricated air-filled components, showcasing the viability of more complex AFSIW component, such as filters, and comparing the proposed solution to the current state of the art at the component and the technology level.

**Table 1:** Overview of D-band technology platforms.

TECHNOLOGY*	Cost	Feature size	Tol.	SR	Scalability	MMIC int.	IL (dB/mm)
CNC^12–15^	High	$<10\,\upmu$m	$2.5\,\upmu$m	Medium$^+$	Low	Complex	0.007^15^
DMLS / SLM^18–22^	Medium	$<50\,\upmu$m	$<15\,\upmu$m	High$^+$	Low	Complex	0.019^18^
SLA^22, 23^	High	$<10\,\upmu$m	$<10\,\upmu$m	High$^+$	Low	Complex	0.025^22^
Injection molding^27, 28^	High	$<10\,\upmu$m	$25\,\upmu$m	High$^+$	High	Complex	0.042$\ddag$^27^
SU-8^13, 14, 31^	Medium	$<1\,\upmu$m	$10\,\upmu$m	Low	Medium	Medium	0.011^31^
SMW^14, 33, 34^	Medium	$<1\,\upmu$m	$<1\,\upmu$m	Low	High	Medium	0.01^34^
LTCC^40, 41^	Low	$25\,\upmu$m	$10\,\upmu$m	Medium	High	Easy	0.4^41^
PCB (this work)	Low	$25\,\upmu$m	$10\,\upmu$m	Medium	High	Easy	0.08

*The minimal feature size and tolerances (Tol.) are highly dependent on the chosen materials, the selected fabrication process and the surface finish.

SR is the surface roughness, $^+$ SR can significantly be improved upon by employing (expensive) smoothing techniques, such as polishing.

IL is the lowest reported mean value of the insertion loss over the entire D-band, except for $\ddag$ evaluated at 110 GHz.

## System technology

**Figure 1:**
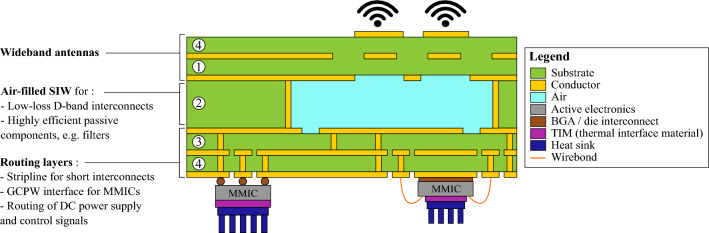
Conceptual representation of the Any-Layer High Density Interconnect (HDI) PCB technology platform with air-filled substrate-integrated-waveguide (AFSIW) layer for high-efficiency D-band wireless systems.

Future system platform implementations need to minimize signal insertion loss when distributing high-frequency signals in large-scale active antenna arrays, while maintaining good power handling. Therefore, we implement air cavities in the middle layer of the PCB stack, as illustrated in Fig. 1, to realize waveguides, filters, antenna cavities and other essential components in a low-loss way, by eliminating dielectric losses. Besides this highly efficient AFSIW layer, which may be used, among others, for signal routing, a low-loss transition to GCPW is present for integration of active devices and measurement purposes^50^. Moreover, a symmetrical stack is proposed for the ease of manufacturing and to reduce the thermal stress when mounting active electronics.

The Any-Layer HDI PCB technology, forming the starting point of our proposed technology platform, is a new generation of the standard HDI PCB technology^51^ that enables the stacking of lasered microvias^52^. This allows connecting each individual layer, enhancing the routing possibilities, along with improved fabrication resolution^53^. We build further up on the Any-Layer HDI PCB technology by including an AFSIW layer, to alleviate the routing loss of this technology, whilst maintaining equivalent fabrication processes^54^ to guarantee compliance with the standard^55^. In particular, the incorporation of the AFSIW layer involves following steps. First, a core laminate $\textcircled {1}$ is patterned, followed by the attachment of a thick prepreg laminate $\textcircled {2}$, in which the AFSIW is lasered and edge-plated. Thereafter, another patterned core layer $\textcircled {3}$, of the same thickness as the first layer $\textcircled {1}$, is attached by means of a sintering process after correct alignment w.r.t. the first two layers^55^. After eliminating dielectric losses, this approach further reduces insertion loss by minimizing the surface roughness of the top and bottom walls of the air-filled components. As the electromagnetic fields propagate in the air-filled region, the surface roughness of the copper foil at the copper/air-cavity interface needs to be considered, in contrast to conventional dielectric-filled substrate-integrated-waveguide (DFSIW) structures where the surface roughness at the conductor/substrate interface needs to be taken into account. The surface roughness at the conductor/substrate interface is typically substantially larger than the surface roughness of the copper foils at the conductor/air-cavity interface to ensure good adhesion of the copper foil to the laminate^56^. To maintain symmetry, each additional prepreg layer $\textcircled {4}$ is added by aligning and pressing^51^ on both sides of the current PCB stack and with equal thickness until the required amount of layers is achieved for the envisaged application. The top layers, above the laminate containing the AFSIW $\textcircled {2}$, facilitate the integration of wideband antenna topologies. The bottom layers enable the routing towards the integrated circuit (IC), including heat sinks to subdue thermal issues. Note the trade-off considered when fixing the thickness of the AFSIW layer: a higher cavity may exhibit a higher Q-factor, whereas a lower cavity enables essentially lower loss in terms of transition towards surrounding layers. Here, we focus on the development of a test vehicle to validate the performance of the proposed manufacturing process, demonstrating its potential as cost-effective alternative to SMW platforms for future sub-terahertz systems.

## AFSIW components

The adopted Any-Layer HDI PCB stack is presented in this section, followed by an in-depth analysis of the AFSIW transmission line, the transition from stripline to AFSIW, employed for measurements and MMIC integration prospects, and the AFSIW dual rectangular cavity filter.

### PCB Stack

**Figure 2:**
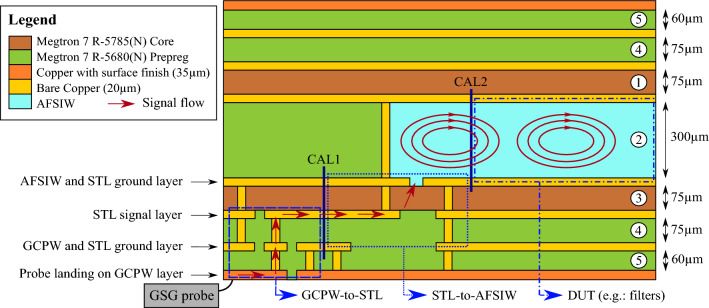
Adopted PCB stack, where the top four layers are intended for future antenna array implementations. A ground–signal–ground (GSG) probe allows measuring the device under test (DUT), whereas the grounded coplanar waveguide (GCPW) to stripline (STL) transition and the STL to air-filled substrate-integrated-waveguide (AFSIW) transition allow calibration up to two different port planes (CAL1 and CAL2).

All aforementioned considerations of the system technology are taken into account when dimensioning the proposed PCB stack. The thickness of each layer is indicated on the right of Fig. 2, together with the order in which the layers are added. The employed process warrants fabrication tolerances down to 10 $\upmu$m for the smallest AFSIW features, such as inductive irises. However, reliable laser-milling and edge-plating imposes a minimal protrusion width of 0.25 mm. This criterion is equally employed as diameter to round off all sharp corners. The features implemented through etching on the conductor layers are precisely manufactured up to 10 $\upmu$m.

Two transitions, transmission lines of different lengths and more complex AFSIW structures, such as filters, are designed following the aforementioned guidelines to validate this PCB stack. For measurement purposes, a GCPW landing pad is currently implemented as an interface to a ground–signal–ground (GSG) probe, which connects to a short GCPW section. In a later stage, these landing pads are to be replaced by MMICs. This results in the signal flows indicated by the red arrows in Fig. 2. A first transition from the short GCPW section to the stripline, integrated on laminates $\textcircled {3}$ and $\textcircled {4}$, allows initial, very compact routing, splitting and combining of the RF signals. The stripline interconnect transitions into the AFSIW components through aperture coupling, which represents the second transition. To extract the maximum amount of characterization data when validating this stack, two additional thru-reflect-line (TRL) calibration sets^57^ are added: one de-embeds up to the stripline section (indicated by CAL1 in Fig. 2), and the other shifts the port plane into the AFSIW transmission line (indicated by CAL2). To this end, each calibration set consists of three standards: a thru, a short that acts as reflect, and a line of which the phase difference with the thru standard remains within [20$^\circ$, 160$^\circ$] to ensure unambiguous TRL calibration.

### Air-filled substrate-integrated-waveguide

**Figure 3:**
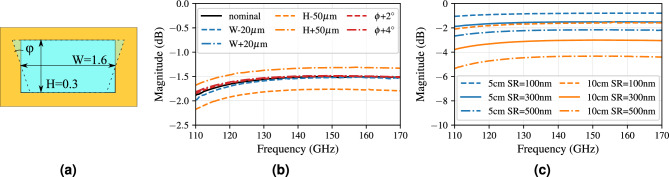
(**a**) The cross-section of the AFSIW transmission lines with indicated dimensions in millimeter. The influence on the transmission coefficient due to (**b**) variations in height (H), width (W) and slant sidewall angle ($\varphi$) for a length of 5 cm, and, (**c**) the surface roughness variations for line lengths of 5 cm and 10 cm.

In this section, the AFSIW transmission lines of two different lengths are analyzed, through full-wave simulations in CST Microwave Studio, based on the cross-section in Fig. 3a. Simulations of the 5 cm-long transmission line, which corresponds to $23.35\lambda _{140\, \text {GHz}, 0}$ in terms of the free-space wavelength at 140 GHz, in Fig. 3b, c, show robust behavior against a change in width or sidewall angle. However, an AFSIW height increase yields an improvement in terms of insertion loss, as can be observed in Fig. 3b. The major influence of the surface roughness is shown for two line lengths, 5 cm and 10 cm, in Fig. 3c. As expected, we find that the insertion loss doubles for the 10 cm-line when compared to the 5 cm-line. Moreover, each 200 nm increase in surface roughness gives rise to an additional 0.01 dB/mm insertion loss. The reflection coefficient remains below − 40 dB for all simulated AFSIW lines.

### Transition from stripline to air-filled substrate-integrated-waveguide

**Figure 4:**
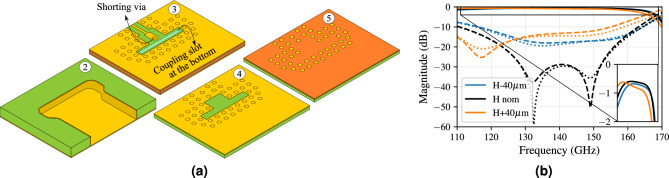
(**a**) AFSIW-to-stripline transition layout^50^, (**b**) influence of AFSIW height variations on scattering parameters (dashed |S$_{11}|$, solid |S$_{12}|$ = |S$_{21}|$ and dotted |S$_{22}|$).

A transition from AFSIW to stripline is designed to supply compact routing features on the one hand, but more importantly, to enable the integration of active electronics and measurements on the other hand. The robustness of the design is ensured for misalignments up to 20 $\upmu$m between the laminate containing the coupling slot $\textcircled {3}$, and the laminate containing the AFSIW $\textcircled {2}$, resulting in a variation in insertion loss smaller than ± 0.02 dB over the entire D-band. To minimize transition loss and ensure maximal routing flexibility, it is important to keep the transition length as short as possible. However, the minimal transition length is restricted by the minimal technologically achievable distance between the shorting via at the end of the stripline and the coupling slot towards the AFSIW. The proposed design^50^ exhibits a transition length of only 0.775 mm, which is equivalent to $0.36\lambda _{140 \,\text {GHz}, 0}$ in terms of the free-space wavelength at 140 GHz. The edge-plated lasercut laminates and the patterning of all relevant conductors in the proposed PCB stack are illustrated in Fig. 4a, employing the same color coding and build up as in Fig. 2.

It is important to analyze the influence of the height of laminate $\textcircled {3}$, containing the AFSIW components. The resulting S-parameters for height variations of ± 40 $\upmu$m are presented in Fig. 4b, showing a degradation in matching as soon as a height deviation occurs, since coupling of the fields into the AFSIW becomes more troublesome. The larger this deviation, the worse the matching, which also leads to an increased insertion loss, indicated in the zoomed part of Fig. 4b. After identification of the actual height, this can be corrected for by re-optimizing the shorting position of the stripline and the coupling slot dimensions towards the AFSIW to improve the matching.

### Filter design

The fabrication of more intricate structures, including protrusions and varying cavity dimensions, are included in a representative D-band system design to validate the proposed PCB stack. A proof of concept consists of a dual rectangular cavity filter, since filters are important elements in the next-generation systems for the exclusion of spurious signals and the reduction of noise. A bandwidth of 6 GHz centered around 135 GHz^58^ is targeted, with a flat transmission response in which the insertion loss remains smaller than 1 dB. In this section, the corresponding theoretical coupling matrix model is compared to a full-wave simulated 3D model of the proposed dual rectangular cavity filter. A tolerance analysis on the crucial properties of the proposed filter is performed to ensure design robustness against the anticipated fabrication inaccuracies.

#### Filter architecture

**Figure 5:**
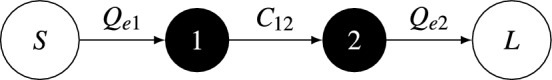
Diagram of the proposed filter.

**Figure 6:**
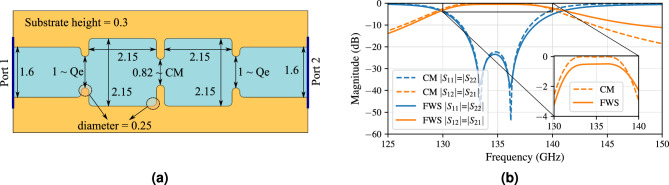
(**a**) Design layout in full-wave simulator CST Microwave Studio, with annotated dimensions in millimeter. (**b**) Comparison of theoretical coupling matrix (CM) generation and full-wave simulation (FWS) of the proposed filter.

 To account for fabrication tolerances, we extend the target 6 GHz operational bandwidth around the system’s center frequency with a guard band of at least 1 GHz, resulting in a bandwidth of minimally 8 GHz centered around 135 GHz. To this end, the envisaged second-order Chebychev filter characteristic is created using coupling matrix theory^59,60^, represented by a simple filter diagram in Fig. 5. The appurtenant external coupling factors ($Q_e$) and coupling matrix (*CM*) for the designed filter are given in equation (1). The scattering parameters pertaining to this theoretical analysis are plotted by dashed lines in Fig. 6b. The minimal insertion loss is trivial: since this is a lossless theoretical model, it amounts to 0 dB. The 3 dB-bandwidth of the transmission characteristic (|S$_{12}|$=|S$_{21}|$) equals 10.2 GHz, ranging from 129.8 to 140 GHz. The transmission exhibits a very flat response, which can be observed in the zoomed in part of Fig. 6b. The filter’s reflection coefficient (|S$_{11}|$=|S$_{22}|$) is matched from 131.7 to 138 GHz, resulting in a fractional bandwidth (FBW) of 4.67%.

1 $$\begin{aligned} Qe = \left[ {\begin{array}{cc} 20 \\ 20 \\ \end{array} } \right] CM = \left[ {\begin{array}{cc} 0 &{} 0.054 \\ 0.054 &{} 0 \\ \end{array} } \right] \end{aligned}$$

#### Full-wave simulations

 Consequently, the theoretical matrices are converted into a physical design, of which the optimized dimensions, based on full-wave simulations in CST, are shown in Fig. 6a. This design takes into account the minimal width of 0.25 mm-wide protrusions imposed by fabrication restrictions. By relying solely on two cavities, the design fits within a footprint of only 5.2 mm $\times$ 2.2 mm $\times$ 300 $\upmu$m. The iris width between the AFSIW feed and the cavity controls the external coupling factor $Q_e$, while the iris width between both cavities is responsible for the coupling coefficient $CM_{ij}$ between both resonators. This design does not suffer from any self-coupling of the cavities ($CM_{ii}=0$).

In Fig. 6b, the resulting S-parameters (solid lines) are compared to the curves obtained from the theoretically conceived filter (dashed lines). The full-wave simulation is carried out with a realistic surface roughness of 300 nm. The deviations w.r.t. the coupling matrix theory, represented by some asymmetry found in the full-wave simulation at higher frequencies, is caused by the propagation of modes with orders higher than the fundamental $TE_{10}$-mode. The minimal insertion loss amounts to 0.48 dB. The 3 dB-bandwidth of 11.2 GHz for the transmission coefficient, ranging from 129.9 to 141.1 GHz, maintains the flat response predicted by the coupling matrix model. The filter’s reflection coefficient is matched from 131.5 to 138.4 GHz, resulting in a FBW of 5.11%. Employing the current dimensions to calculate the quality factor for a single cavity^61^, resulting in $Q_{theory, single} = 975.5$, allows comparison to the full-wave simulated equivalent, being $Q_{CST, single} = 1393.2$. However, neither the measured surface roughness nor the employed surface finish of the fabricated prototype is taken into account in these calculations, while their importance will be confirmed later in this paper.

#### Fabrication cornerstone analysis

**Figure 7:**
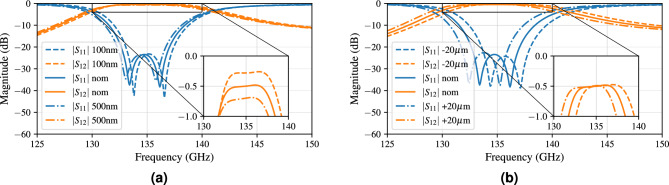
Influence of (**a**) surface roughness variations, (**b**) a variation of 20 $\upmu$m on the cavity dimensions.

 This analysis investigates three important fabrication imperfections, being (1) the influence of the surface roughness, (2) the variation of several key dimensions of the dual rectangular filter, and (3) the potentially slant sidewall angle of the AFSIW cross-sections. A moderate change in surface roughness on the sidewalls of the AFSIW barely has any consequences, since the fields are contained in the center of the structure. Conversely, a variation on the surface roughness on the top and bottom conductors of the AFSIW results mostly in an elevated insertion loss and a slight shift towards lower frequencies due to the increase in path length. This is indicated in Fig. 7a for a surface roughness of 100 nm, 300 nm (nominal) and 500 nm. Each 200 nm increase in surface roughness causes 0.2 dB additional insertion loss. This shows the importance of smooth copper foils when fabricating the AFSIW components, to keep the overall insertion losses in future communication systems to a bare minimum.

The subsequent key dimensions of the design are tightly related to the operating mechanism of this filter. The cavity dimensions determine the resonant frequencies and, therefore, the passband location. By decreasing the cavity dimensions, the resonances will shift towards higher frequencies, as will the passband. This behavior can be observed from Fig. 7b, where the cavity dimension varies from 2.13 mm, 2.15 mm (nominal) to 2.17 mm. Each joint length and width increase of 20 $\upmu$m results in a 1 GHz-decrease in operating frequency, neatly indicating our previously defined guard bands around the operating frequencies.

**Figure 8:**
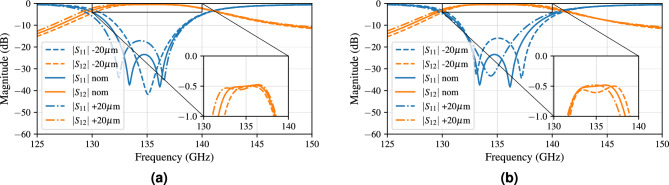
Influence of 20 $\upmu$m variations on (**a**) the coupling coefficient ($CM_{12}=CM_{21}$) linked to the iris width between the cavities, (**b**) the external coupling (*Qe*) linked to the iris width between the AFSIW feed and the cavity resonator.

The coupling matrix coefficients ($CM_{12}=CM_{21}$) are directly affected by changing the iris width between both cavities. Increasing the iris width increases the coupling between both cavities, which is confirmed in Fig. 8a. When the cavities are too tightly coupled, the passband is deprived of its flat characteristic, since the resonances shift too far away from each other. This is illustrated for an iris width of 0.84 mm in Fig. 8a (dashed-dotted line). On the other hand, the iris width of 0.8 mm decouples the cavities, bringing the resonances so close together that the passband is greatly reduced (dashed line). Ultimately, an optimum can be found to obtain the desired flatness and operational bandwidth. The external loading factors (*Qe*) are directly affected by changing the iris widths between the incoming AFSIW feed and the neighboring cavity. Figure 8b shows that an increase of this iris width brings the resonances closer together and lowers the passband very slightly in frequency.

**Figure 9:**
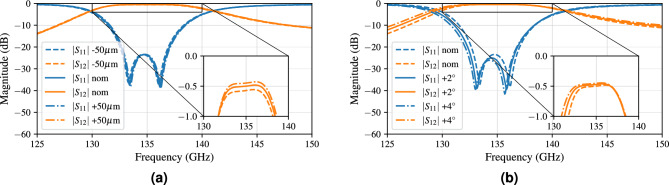
Influence of (**a**) the height of the AFSIW layer, (**b**) the angle of the sidewalls.

The increase in AFSIW height, as stated previously, improves the Q-factor^61^. Figure 9a corroborates this improvement by showing lower insertion loss for increasing heights. The last possible fabrication deviation occurs at the sidewalls of the AFSIW. The sidewalls may be slant due to laser beam diffusion when fabricating the AFSIW segments. The resulting effect on the filter characteristic is demonstrated in Fig. 9b, proving robust behavior against minor variations in sidewall angle. A 2$^\circ$ increase in sidewall angle results in a shift towards lower frequencies by less than 0.2 GHz for both resonances. The simulated slant angles are defined by rotating the sidewalls around their height-center. Therefore, a height of 300 $\upmu$m gives rise to a wall location variation of ±  5 $\upmu$m per 2$^\circ$ increase in sidewall angle. In case of a 2$^\circ$ sidewall angle, the width of the cavity dimension varies in the range 2.15 mm ± 10 $\mu$m.

## Measurement results

### AFSIW routing

**Figure 10:**
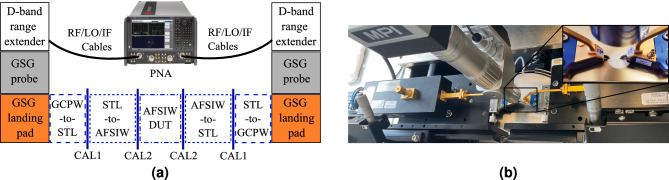
(**a**) Block diagram of the measurement setup with indicated calibration planes. (**b**) Corresponding measurement setup.

 The first validation step of the suggested PCB technology platform, presented in Fig. 2, consists of measuring the double transition with two AFSIW lines of different length: 5 cm and 10 cm. These measurements are performed by attaching the Infinity Waveguide Probes (with a GSG pitch of 100 $\upmu$m) to the Tx/Rx VDI Extension Modules WR6.5 (110–170 GHz). These range extenders are connected to the Keysight N5247B PNA-X vector network analyzer (VNA), as schematically explained in Fig. 10a, according to the annotations in Fig. 2 to clarify each step. An MPI microscope is employed to validate the positioning of the probes on the GCPW landing pads, as shown in the actual measurement setup in Fig. 10b.

**Figure 11:**
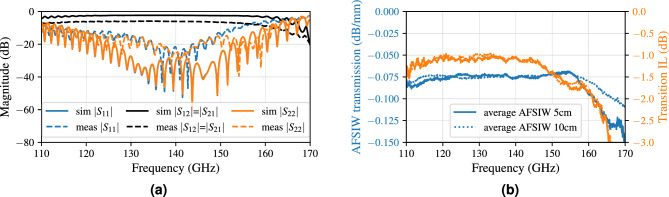
(**a**) Comparison of simulation (sim) and measurement (meas) of a 5 cm-long AFSIW transmission line including transitions towards stripline (CAL1). (**b**) The transmission loss per millimeter of the AFSIW waveguide and the insertion loss (IL) of the stripline-to-AFSIW transition over the entire D-band.

The acquired data are post-processed using the Python scikit-rf package for TRL calibration^57^. Two separate calibrations are executed up to the port planes marked in Fig. 10a, called CAL1 and CAL2. The measurement deembedded up to CAL2 yields the characteristic of an electromagnetic wave traveling through a purely AFSIW section, while the CAL1 calibration planes include two transitions leading up to the striplines. The CAL1 calibration for the 5 cm-long AFSIW line is plotted in Fig. 11a, comparing our simulation model to the measured result. Good correspondence between both can be observed. The repeatability of the fabrication is validated by measuring three prototypes and further processing is done on the average of the transmission characteristics of all measurements. Comparing both CAL1 and CAL2 data, of which the corresponding curves are plotted in Fig. 11b, shows a 1.1 dB-transition loss from stripline to AFSIW. Figure 11b also demonstrates that the AFSIW interconnect has a measured insertion loss of around 0.08 dB/mm in the D-band and an amplitude variation smaller than 0.01 dB/mm from 115 to 155 GHz.

Table 2 compares the implementation of different routing options in different fabrication technologies. The DFSIW, microstrip line (MSL) or GCPW suffer from a high dielectric loss, which corresponds to an increased loss per unit length. Other AFSIW options, such as CNC milling, 3D printed SLA or SLM and SMW, outperform our proposed solution in terms of insertion loss, owing to lower surface roughness at the conductor–air interface and/or higher waveguides. Nevertheless, they face several challenges associated with MMIC integration^11,62^. To this end, our proposed solution delivers a cost-efficient, mass-manufacturable alternative that facilitates integration of MMICs. Future research concerning the optimization of the fabrication procedure might decrease the surface roughness further and/or increase the height of the AFSIW section, thereby, decreasing the insertion loss of our proposed solution. While showcasing the importance of the conductor foils with low surface roughness, our results compete with other AFSIW-containing technologies as a cost-efficient, mass-manufacturable alternative that promotes integration of MMICs.

**Table 2:** Transmission line losses at sub-terahertz frequencies for different implementation technologies.

Technology	Freq. (GHz)	Loss (dB/mm)
CNC milled RWG^15^	110–170	0.005–0.008
SLM 3D-print RWG^18^	110–170	0.014–0.023
SLA 3D-print RWG^22^	120–170	0.01–0.04
KOH SMW^63^	75–110	0.013
DRIE SMW^34^	110–170	0.007–0.013
SIW on silicon^62^	110–170	0.4–0.6
GCPW on PCB^5^	110–170	0.123–0.216
GCPW on LTCC^41^	110–170	0.3–0.4
MSL on PCB^5^	110–170	0.132–0.238
MSL on LCP^64^	110–170	0.176–0.331
MSL on BCB^11^	110–170	0.6–0.8
PCB (this work)	115–155	0.07–0.08

### AFSIW filter

**Figure 12:**
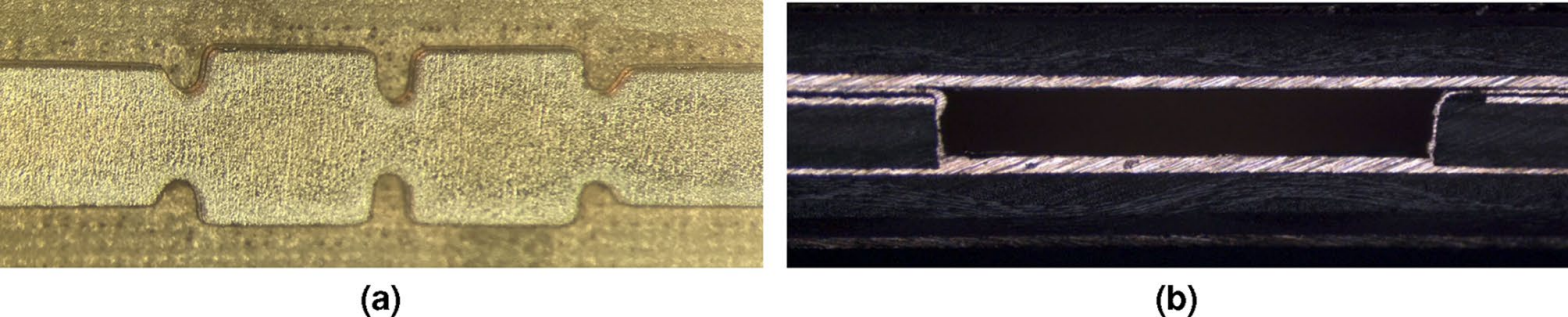
Fabricated prototype (**a**) top view cross-section, (**b**) PCB stack cross-section.

 To illustrate the technology’s potential for the development of high-performance functional components in the proposed Any-Layer HDI PCB stack, besides routing components, we now characterize the dual rectangular cavity filter, realized in this proof of concept. It consists of the layout represented in Fig. 6a, which is directly connected to the AFSIW-to-stripline transition for measurement purposes. A top view of the fabricated AFSIW filter is shown in Fig. 12a before the third laminate $\textcircled {3}$ is added, as explained previously. A cross-section of the AFSIW feed is shown in Fig. 12b, indicating a slight inclination of the sidewalls, of which the effect should be negligible, as discussed during the tolerance analysis.

**Figure 13:**
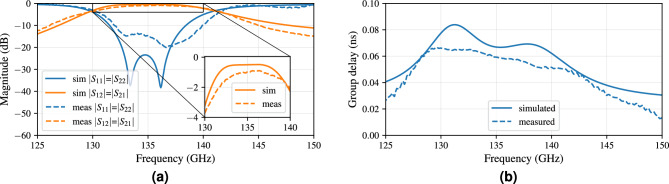
(**a**) Comparison of full-wave simulation and measurement. (**b**) Group delay of the filter.

The corresponding S-parameters in Fig. 13a are in good agreement with the simulations. The minimal insertion loss of the measured filter is 0.87 dB. The target operating bandwidth of 6 GHz around 135 GHz is achieved, since the measured 10 dB-match spans from 132.8 to 139.2 GHz, which corresponds to a FBW of 4.74%. The 3 dB-bandwidth covers 10.3 GHz, ranging from 130.6 to 140.9 GHz. In contrast to the aforementioned quality factor calculations, more realistic Q-factors can be obtained based on the scattering characteristics^61^. In simulation, we obtain a value of $Q_{sim,filter} = 236$, while the measurements yield a value of $Q_{meas,filter} = 153$. The decreased measured value is expected, since the higher insertion loss with respect to the simulation is not compensated by the smaller 3 dB-bandwidth^61^. Figure 13b show the maximal measured group delay of 0.067 ns and its simulated equivalent of 0.084 ns. The filter provides a solicited flat group delay within the passband, competing with literature^65,66^.

All high-end D-band filters in the state-of-the-art literature are fabricated in AFSIW technology to minimize the routing losses and maximize power throughput. Table 3 compares some high-end filters implemented in DFSIW and AFSIW based on different fabrication procedures to our proposed solution. The LTCC prototype shows an excellent footprint, but has limited FBW and suffers from large transmission losses due to the prominent presence of a dielectric laminate. Comparing the CNC and DMLS prototypes to each other, reveals an increased insertion loss due to the surface roughness in case of DMLS 3D-printing. The SMW filter yields an excellent compromise between the latter two alternatives, further enabling integration of MMICs and parallel mass-manufacturing to suppress the cost. Our proposed PCB filter is compact for an AFSIW implementation, owing to the dual-cavity topology and, therefore, maintains a broadband characteristic and a low insertion loss. The validation of this filter characteristic paves the way to the design of more complex filters, which will be in competition with the current state of the art, as predicted by Table 3.

**Table 3:** Comparison of the proposed solution with the state-of-the-art designs.

Technology*	Order	Size (mm$^2$)	$\Delta$f$_0$ (GHz)	FBW (%)	IL$_{min}$ (dB)	RL (dB)
CNC^67^	5	$\approx$ 13 $\times$ 8	130–134/151.5–155.5	4.30	0.5	20
DMLS^67^	5	$\approx$ 13 $\times$ 8	130–134/151.5–155.5	4.30	1.35	8
SMW^68^	6	9.4 $\times$ 2	130–134	5	1.2	20
LTCC^69^	4	3.32 $\times$ 2.37	148.7–150.7	1.30	5.5	10
PCB (this work)	2	5.2 $\times$ 2.2	131–138	8.70	0.86	10

* $\Delta$f$_0$ is the frequency range, FBW is the fractional bandwidth, IL$_{min}$ is the minimal insertion loss and RL is the return loss.

## Conclusion

This paper presents a versatile, mass-producible Any-Layer High Density Interconnect (HDI) printed circuit board (PCB) technology for future joint communication and sensing (JCAS) systems at D-band (110–170 GHz). The addition of a low-loss air-filled substrate-integrated-waveguide (AFSIW) layer, for highly efficient D-band routing and filtering, helps to overcome the current shortcomings of PCB technology by avoiding dielectric losses and minimizing losses due to surface roughness. A dedicated transition from AFSIW to stripline is designed for compact and broadband interfacing to integrated circuits (ICs), accentuating an additional advantage of PCB technology. To this end, several AFSIW components, including an AFSIW-to-stripline transition, are analyzed and their measured characteristics are validated with respect to simulated data. We demonstrate that the measured air-filled routing loss is around 0.08 dB/mm, while ensuring a flat amplitude variation within 0.01 dB/mm over a broad range (115–155 GHz). The manufactured transition towards stripline reveals a loss of only 1.1 dB. The fabricated dual rectangular filter has a minimal insertion loss of 0.87 dB and exhibits 10 dB-matching within the (132.8–139.2 GHz) frequency band, while maintaining a good quality factor of 153 and a group delay not exceeding 0.067 ns. Moreover, the insertion loss can be further improved in future generations by reducing the surface roughness of the conductor layers at the AFSIW boundaries and/or further increasing the AFSIW height. To this end, additional research is to be performed for optimization of the fabrication procedure. The good performance of the measured prototypes showcase the potential of this novel PCB-based technology platform, thereby paving the way for the development of full system front-ends consisting of chip integration, routing, filtering and radiating features, alleviating future obstacles towards the next-generation wireless systems.

## Data Availability

The datasets used and analyzed during the current study available from the corresponding author on reasonable request.
